# Like Father, Like Son. Physical Activity, Dietary Intake, and Media Consumption in Pre-School-Aged Children

**DOI:** 10.3390/ijerph16030306

**Published:** 2019-01-23

**Authors:** Nadja Frate, Brigitte Jenull, Robert Birnbacher

**Affiliations:** 1 Department of Psychology, Alpen-Adria-Universität Klagenfurt, 9020 Klagenfurt, Austria; nadja.frate@aau.at; 2 Department of Children and Adolescent Medicine, Hospital Villach, 9500 Villach, Austria; Robert.Birnbacher@kabeg.at

**Keywords:** preschool children, physical activity, dietary intake, recommendations by the WHO, media consumption

## Abstract

An imbalance between energy input and energy needs contributes to the growing incidence of overweight children. Pre-schoolers normally like to move, but even at this young age, they are already affected by a lack of physical activity and a high amount of screen time. Media consumption contributes to unhealthy diets and extends the length of time spent sitting. Longer periods of sitting are, independent of the level of activity, seen as a risk factor for the development of obesity. In the present study, 160 pre-schoolers and their parents (128 mothers, 121 fathers) were examined. The results show deviations from actual recommendations regarding physical activity, time spent sitting, dietary intake, and media consumption. Increased screen time was associated with a higher weight status among pre-school-aged children. To provide a healthy upbringing, prevention and intervention measures should be implemented on a behavioral and relational level.

## 1. Introduction

The prevalence of obesity has more than doubled in the last 35 years. In 2014, approximately 41 million children under 5 years of age and 1.9 billion adults were overweight or obese [1]. In addition to social factors (income, education, media consumption) and psychological factors (attachment, body satisfaction), biological factors in particular (nutrition, exercise) play a role in the development of overweight [2].

According to Huber [3], the main mechanism triggering weight gain is an imbalance of energy input and energy need: High food energy intake is accompanied by low levels of physical activity, long periods of time spent sitting and high amounts of screen time [4,5]. By means of the metabolic equivalent of task (MET), the energy consumption between inactivity and activity can be made clear. One MET is 4.2 KJ (1 kcal) per kg body mass/hour and approximately equals the resting metabolic rate of a human [6]. When one is sitting, only 1–1.5 MET are used. Mild physical activity consumes 1.5–3 METs. Moderate physical activity (activity that leads one to be slightly out of breath) consumes 3–6 METs, and strong physical activity consumes over 6 METs [6,7].

To counter becoming overweight, one must engage in enough activity. International physical activity recommendations by the WHO suggest engaging in at least 60 min of activity per day with moderate intensity [8]. Worldwide, only a small number of pupils reach these suggestions for physical activity [9]. Tucker [10] investigated the physical activity of pre-schoolers in a review. The recommendation of 60 min of moderate- to high-intensity activity by the National Association for Sport and Physical Education (NASP) [11] was not reached in 46% of studies. During Wave 1 of the German Health Interview and Examination Survey of Adolescents and Children (KiGGS), 27.5% of the 10,426 children aged 3 to 17 years reached the WHO recommendations for physical activity [12].

Data of the participants in the Health Behaviour in School-Aged Children (HBSC) study from 32 countries showed that younger age, male gender and socioeconomic status were positively correlated with physical activity time [13].

Cooper et al. [14] showed that after the age of six, physical activity reduces by 4.2% per year. This result has also been confirmed by other studies [15,16,17], whereas time spent inactive or sitting increases from childhood into youth and adulthood [15,18]. Studies from Portugal, the Netherlands, and the USA concluded that 2- to 6-year-old children spend 50% to over 80% of their day in sitting activities [19,20,21,22]. Experts recommend limiting sitting times to a maximum of two hours per day [23].

Inactivity or lack of activity at younger ages is related to higher media consumption [7]. Media consumption includes watching TV, using a computer (e.g., social media, games) or playing with smartphones or game consoles. Media consumption often begins as soon as in early childhood. The age of first television consumption was five months in 2007. Four decades earlier, children first watched television at the age of four [24]. Among pre-school-aged children 2 to 5 years old, 50–80% watched television for more than one hour per day [20,25,26].

The Identification and Prevention of Dietary and Lifestyle-Induced Health Effects in Children and Infants (IDEFICS) study found that for 3604 children aged two to six years, higher media consumption predicts lower well-being longitudinally [27]. Mendoza, Zimmerman, and Christakis [28] found connections between high media consumption and a risk of obesity in children as young as ages two to five.

Regarding dietary intake, studies indicate a connection between a high sugar intake, mainly through sweets and soft drinks [29,30], and the development of obesity in childhood. In addition, unhealthy snacks and large portions among pre-school-age children are considered risk factors for developing obesity [31]. Watching TV and being in front of the PC favor unhealthy nutrition and higher energy intake [32]. Several studies indicate that TV viewing influences the intake of sweets and soft drinks [33,34,35]. Aue and Huber [36] note that, on the one hand, children tend to be inattentive while consuming media and, therefore, do not recognize that they are already full [37]; on the other hand, snacking could explain these results.

The purpose of this study is to better understand the relevant factors for childhood obesity in a rural region in Austria, by focusing on the relationship between physical activity, dietary intake, and media consumption of pre-school children and those of their parents.

## 2. Materials and Methods

The target group of the survey consisted of 160 pre-school children attending kindergarten (from 10 kindergartens with half-day and full-time care) and their parents. We obtained our data from the DONUT-Project (https://campus.aau.at/cris/project/0f4de0c75c9c2c91015ca09df4bc01cf), a long-term study examining the bio-psychosocial risk factors of childhood obesity. In this research, the main method was a survey based on a questionnaire.

### 2.1. Demographics

Parents completed a demographic questionnaire that included questions about their citizenship, monthly household income and composition, household size, highest level of education, occupational group, and time spent working per week. With this information, we calculated the weighted net equivalence income according to the Organisation for Economic Co-operation and Development (OECD) scale [38] and socioeconomic status (SES), a multidimensional index computed as the sum score of points from the parental data [39]. With this information, a classification of low, medium, and high social status became possible.

Additionally, parents reported their body weight, body height, and age, as well as their child’s age. Parental BMI was categorized as follows: BMI ≥ 25.0 kg/m^2^ to 29.9 kg/m^2^ as overweight and BMI ≥ 30 kg/m^2^ as obese. Trained study staff measured each participating child’s height and weight in the kindergarten classrooms using a calibrated SECA 877 scale and a portable stadiometer. Measurements were taken while the child was in light clothing and no shoes. Age-specific percentile values were calculated using the Centers for Disease Control (CDC) [40] guidelines. Children who had a BMI percentile between the 85th and 95th percentiles for age and sex, were classified as overweight, and those at, or above, the 95th percentile were classified as obese.

### 2.2. Physical Activity

Physical activity was measured using the International Physical Activity Questionnaire (IPAQ, https://sites.google.com/site/theipaq/home). The instrument contains questions about the activity behavior of adults, within the last seven days in four areas (time spent sitting, time spent walking, moderate activity, and high activity), which were summarized into overall activity in MET-minutes/week. According to the adult version of IPAQ, we formulated questions for children’s physical activity that were answered by parents. For example: How much time does your child usually spend each day doing everyday routines or walking? The answer was given in hours and minutes. Based on the information, the activity behavior can be divided into three activity levels: low, moderate, and high.

### 2.3. Dietary Intake

The parents recorded foods and beverages that they usually consumed. Detailed instructions with pictures were provided according the WHO recommendations [1] to aid in the estimation of the quantity of food by children and parents. For example: In how many of the past 7 days have you and your child eaten fruits? A portion of fruit equals the amount that can be stored in your own hands. For smaller fruits, two hands make one portion. A portion can also consist of a glass of 100% fruit juice. Responses are given as days in a week and the number of portions per day. All information was used to categorize each participant´s total intake into food groups (sweets, fast food, alcohol/fats and oil/fish, meat, sausage, eggs/milk products/wheat and potatoes/fruits, and vegetables/water).

### 2.4. Media Consumption

Media use included both TV and computer exposure among the children and their parents. For example: How many hours a day does your child play with computers and/or game consoles? Responses were given in hours and minutes. Furthermore, parents answered questions about the number of televisions in the household, their average sleep times and those of their children.

### 2.5. Analysis

Due to an exploratory design, our study was mainly analyzed descriptively. Correlations and linear regressions were calculated in order to find associations between parental and childhood physical activity, dietary intake, and media consumption. For the analyses of differences between high and low media users, we used independent samples *t*-tests. The data were analyzed using IBM SPSS Statistics version 24 (SPSS Inc., Chicago, IL, USA).

### 2.6. Ethical Considerations

The study received approval from the Alpen-Adria-Universität Ethics Committee (2018-028). Participants were informed about the aims and procedure of the project and were assured that participation was voluntary and that they were free to withdraw without stating a reason. They were guaranteed confidentiality and anonymous presentation of the findings. Written consent was obtained.

## 3. Results

### 3.1. Demographics

Overall, there were 160 children participating in the study. The sex composition was balanced, with 51% (*n* = 81) boys and 49% (*n* = 79) girls. The children were five years old on average (*SD* = 1.03). Furthermore, 128 mothers and 121 fathers participated. On average, the mothers were 34 years old (*SD* = 4.73, *n* = 128) and the fathers were 37 years old (*SD* = 6.01, *n* = 121). A total of 121 mothers and 115 fathers had Austrian citizenship. Four mothers and six fathers were from Germany. One family was from Italy, one from Bosnia, and one from Croatia. A total of 123 families lived in cities with less than 5000 inhabitants. The average time spent working per week for the mothers was 23 h (*SD* = 11.22, *n* = 109) and 43 h for the fathers (*SD* = 9.06, *n* = 114). The monthly “equivalent household disposable income” of the participating families was, on average, 1475 Euro (*SD* = 550.62, *n* = 108). In the Austrian comparison, this income is in the lower quartile range [41].

The categorized SES of the sample is reported in Table 1.

### 3.2. Weight Outcomes

The children were, on average, at 47.70 percentile (*SD* = 30.58) for weight. Table 2 provides information about the weight distributions of the children and their parents.

The percentile values of the participating children were highly and significantly correlated with the BMI of their fathers (*r* = 0.288, *p* = 0.002), and were significantly correlated with the mother’s BMI (*r* = 0.177, *p* = 0.046).

The results of a linear regression analysis showed that 8% of the variance of children’s percentile values can be explained by the father’s BMI (*F*(1,113) = 10.238, *p* = 0.002). The BMI value of the mother explains 3.2% of the variance in the children’s percentile values (*F*(1,125) = 4.066, *p*= 0.046). Therefore, the fathers’ and the mothers’ BMI were useful in predicting the children’s BMI percentile.

### 3.3. Physical Activity

Of the 123 children with complete IPAQ data, the vast majority could be classified into the moderate activity level (Table 3). None of the children were categorized as low. The parents were mostly categorized as high.

Eighty percent of the children were classified into the moderate category; for example, they reached at least five days by a combination of vigorous, moderate-intensity activities, or a combination of walking a minimum of at least 600 MET-minutes per week.

Every fifth child and most of the parents were in the high category, which was achieved by vigorous-intensity activity on at least 3 days, achieving a minimum of at least 1500 MET-minutes per week or 3000 MET-minutes per week (IPAQ, https://sites.google.com/site/theipaq/home).

The WHO [8] recommends a daily activity time of at least 60 min with moderate intensity.

Although the majority of children fell into the moderate category, it must be noted critically in our study that the average duration of exercise was 46.5 min (*SD* = 85.17), and only 19% of children moved more than 60 min per day. Approximately 81% did not reach the recommended activity of one hour per day at moderate to high intensity.

### 3.4. Time Spent Sitting

Children sit approximately 188 min per day (*M* = 188.17, *SD* = 95.71, *n* = 123). Mothers spent 270 (*M* = 269.52, *SD* = 144.00, *n* = 124) and fathers spent 314 min sitting (*M* = 313.74, *SD* = 176.39, *n* = 107). A total of 59% (*n* = 73) of children sit more than two hours per day. The daily time spent sitting for the children correlates with the daily time spent sitting of mothers (*r* = 0.312, *p* < 0.001) and fathers (*r* = 0.279, *p* = 0.004), and is correlated with the amount of sweets (*r* = 0.279, *p* = 0.004), and sweetened beverages (*r* = 0.239, *p* = 0.010) children consumed weekly.

### 3.5. Deviations of the WHO-Nutrition Recommendations

Parental data for regular nutrition were compared to the food pyramid guidelines (WHO). Table 4 shows the mean deviations of calories, amount of meals, portions, and milliliters from the nutritional recommendations of the WHO [1].

Mothers’ reported nutritional habits correspond widely with the recommendations. For the fathers, deviations could be found, especially at the top of the food pyramid, as each week there was consumption of about 1000 calories more than recommended in sweets, sweetened beverages, alcohol, or fast food. Moreover, fathers ate 3 ½ more portions in fish, meat, sausage, and eggs than recommended. Children had the greatest discrepancy in the category “sweets” and in the category “water”. They drink, on average 544 mL less water per day, than is recommended.

The caloric proportion of foods from the peak of the nutritional pyramid that the children consumed each week (sum of sweetened beverages, fast food, and sweets) is significantly correlated with the days on which the mothers (*r* = 0.374, *p* < 0.001) and fathers (*r* = 0.304, *p* = 0.002) consumed sweetened beverages. Moreover, there are clearly correlations between the caloric intake of mothers (*r* = 0.699, *p* < 0.001) and fathers (*r* = 0.502, *p* < 0.001) in the form of sweets and fast food (mothers: *r* = 0.406, *p* < 0.001; fathers: *r* = 0.268, *p* = 0.004).

### 3.6. Media Consumption

Nearly half of the 126 families (42.9%) have more televisions in their household than just the one in the living room. One family owned four TVs (2.0%), five families had three TVs (9.8%), 28 families had two TVs (54.9%), and 17 families had one TV (33.3%). Every tenth child in our study had his or her own TV in his or her room (*n* = 18, 11.8%).

The mean TV viewing of the children was nearly one hour (58 min, *SD* = 42.54, *n* = 126). A total of 52% of children watched TV more than one hour per day (*n* = 65; range = 300_max_–60_min_) and can be described as high media users. Mothers watched an average of 104.30 min of TV per day (*SD* = 51.01, *n* = 121), and fathers watched 112.77 min per day (*SD* = 53.90, *n* = 110).

The BMI percentile values of the children correlated significantly with their daily TV viewing (*r* = 0.187, *p* = 0.037). Approximately 3.5% of the variance in the children’s BMI percentile values can be explained by the number of minutes that the children spent watching TV daily (*F*(1,123) = 4.469, *p* = 0.037).

Significant correlations were found between the daily TV viewing of mothers (*r* = 0.238, *p* = 0.002) and fathers (*r* = 0.283, *p* = 0.003), and children’s TV viewing. There was also a significant negative correlation between time spent watching TV in childhood and the level of education of mothers (*r* = −0.221, *p* = 0.014) and fathers (*r* = −0.244, *p* = 0.008).

Among the children who spent more than 58 min per day watching TV, mothers also spent more time watching TV (*M* = 113.95 min, *SD* = 50.34, *n* = 62) in comparison to mothers of children who watched TV for less than 58 min (*M* = 94.30, *SD* = 51.02, *n* = 57), *t*(117)= −2.114, *p* = 0.037. The families of the children who had high TV viewing (over 58 min) also had a lower SES value (*M* = 12.76, *SD* = 3.40, *n* = 51) than families of children with lower TV viewing (*M* = 14.13, *SD* = 3.15, *n* = 51; *t*(103) = 2.1739, *p* = 0.035).

### 3.7. Computer, Telegames and Phone Use

A total of 151 children made statements about the use of computers, tablets, gaming consoles, or mobile phones. Daily computer use correlated positively with the computer use of mothers (*r* = 0.384, *p* < 0.001) and fathers (*r* = 0.330, *p* = 0.003).

Positive correlations were found between computer use and the amount of calories consumed by the children (*r* = 0.222, *p* = 0.028). Correlations were also found between computer use and the number of televisions in the household (*r* = 0.301, *p* = 0.047), as well as the amount of time mothers spent working (*r* = 0.237, *p* = 0.028).

In the *t*-tests, children with computer use significantly differed from those who had no computer use in their percentile values (*t*(149) = −2.04, *p* = 0.043).

## 4. Discussion

The results of the study show that even pre-school-aged children engage in behaviors that are harmful to their health. These behaviors favor an energy imbalance through low physical activity, with a simultaneously high amount of time spent sitting and inactivity by higher media consumption.

The analyses regarding physical activity showed that the majority of the participating children were categorized into the moderate group. No child was assigned to the low-activity category, which can be explained by the high number of minutes of everyday activity. The parents were almost consistently categorized into the high category of physical activity. According to Bauman et al. [42] more than half of the adult population denominate themselves as highly active. The prevalence of high physical activity in our study can be explained by socially desirable responses, which cannot be ruled out in self-reports [43]. Parents influence the behavior of children mainly through their own behavior [44]. An active parental lifestyle leads to more physical activity of children; To the same extent, children mimic their parents’ dietary habits [45,46]. It can be presumed that the parents may be seen as role models who teach their children about having fun in movement and sports. Nevertheless, only every fifth child moves more than the recommended 60 min per day at moderate to high intensity. In contrast to the findings of a systematic review by Tucker [10], that examined the physical activity levels of 10,316 preschool-aged children, slightly more than half of the participants in this study met the recommendation of a minimum of one hour of physical activity per day. One explanation for the discrepancy in physical activity values could be the use of different instruments. The over-estimation of self-reported physical activity has been described elsewhere [47], therefore our IPAQ results must be interpreted with caution.

According to behavioral prevention principles, it would be meaningful to design the children’s familial, as well as communal environment. Children should have enough moving space, incentive and time, which can be used to reach the international recommendations for physical activity. An investment is worthwhile, because physical activity in childhood is often maintained during the lifespan [48,49].

We found associations between children’s and parents’ BMI. The high percentage of overweight and obese fathers in our study could be explained by the sole use of BMI because no information about the body-fat distribution was available. Other measures of body composition, such as hydrostatic weighing or skinfold ratio [50], should be used in future studies.

Our study suggested no consistent associations between children’s physical activity and sex, SES and overweight, as prescribed in other studies [13,51]. This finding may be a function of our sample, which included very young children. Another explanation could be the rurality of the sample, where children from families with low SES find a safe, easy-to-move, and movement-friendly environment.

Although the participating children are raised in a movement-friendly environment with physically active parents, they spent three hours a day sitting. A sitting lifestyle has negative health consequences. In addition to contributing to a higher risk of obesity, it should be noted that sitting also increases the risk of lower bone density [52].

Deviations from the nutrition recommendations of the WHO were found at the top of the food pyramid. Children ingested more than 513 extra calories through sweets, sweetened beverages, and fast food. Furthermore, we found that children drink, on average, 544 mL less water or unsweetened beverages, per day, than is recommended.

In addition to optimal nutrition, it would be desirable to promote children’s competence in choosing food, which could also be encouraged in kindergarten classes [53]. At the same time, the negative influence of food advertisements should be politically questioned. It would be interesting to look at advertisements from the perspectives of preschool-aged children because they are incapable of questioning the intentions of the seller [31]. Parents are mainly responsible for dealing with media. First, screen time should be monitored and critically questioned. Second, the length of consumption time should be regulated. Graf et al. [54] recommend avoiding the placement of a television in a child’s room and limiting the consumption time to a maximum of 30 min among 3- to 6-year-olds. The daily amount of time spent watching TV in this study was approximately one hour [23]. There is a connection between a child’s TV viewing and the TV viewing of his or her parents, which is consistent with previous findings [26,55,56]. Moreover, we found correlations in weight status, as well as in daily TV viewing between children and their parents.

Several studies have shown that an unhealthy nutritional lifestyle is associated with media consumption [29,30,33,34,35]. The authors especially thematized sweetened beverages and sweets.

It is astonishing that two-thirds of the participating children were using a computer, tablet, gaming console, or phone. Children engaging in computer use had higher percentile values than children without computer use. As with TV use, the child’s computer use has a connection to the parent’s computer use.

Our study has some limitations. First, our study’s data were collected by questioning the parents. In addition to the potential limitation of social desirability, which can distort the objectivity of the data collected, physical activity is difficult to measure with questionnaires [43]. Even established instruments, such as the IPAQ can lead to biases such as over- or under-estimations regarding the intensity of the activity [47]. Future studies should target more in-depth exploration of the complex relationship between young children and their parents, to identify relevant factors and initiate a healthy lifestyle [44,45,46]. Second, our sample is from kindergartens within a rural area, which potentially limits the generalizability of the findings to other settings. Finally, the data were obtained from a cross sectional design; thus, there can be no determination of causal relationships, directional associations and interpretations, and changes and differences in the developmental course, mediation processes, or bidirectional effects.

## 5. Conclusions

Prevention programs are more effective in younger children. This finding was confirmed in a meta-analysis by Waters et al. [57], which included 37 controlled studies. Therefore, the ages of the children in our study would be ideal for prevention programs aiming to achieving sustainable changes.

However, the reversal of a low energy expenditure through physical activity seems to be a component in preventing overweight. Some studies have shown that only promoting physical activity is not sufficient [58,59]. An important key to maintaining children’s health lies in reducing sitting time and media consumption.

Within the family setting and in kindergarten classes, prevention measures should mostly concern parents and significant others. The transfer of knowledge and the optimization of activity, dietary intake, and media consumption behavior is particularly important [60]. Through direct contact with children, the interaction of activity, media consumption, and nutrition can be conveyed in a playful way.

Obesity prevention does not concern only parents and kindergartens; rather, it must not be forgotten that public health services and politics need to be synchronized. For example, doctors are in direct contact with parents and can convey important information [61].

## Figures and Tables

**Table 1:** Categories of socioeconomic status (SES).

SES Categorized *n* = 107 *	Frequency
Low (3–8.4)	11 (10.3%)
Moderate (8.5–15.4)	62 (57.9%)
High (15.5–21)	34 (31.8%)

* Data gathered via parent surveys. Sample sizes vary according missing responses.

**Table 2:** BMI of children and their parents.

Weight Status	Children *n* = 159	Mothers *n* = 128	Fathers *n* = 116
Underweight	*n* = 16 (10.1%)	*n* = 6 (4.7%)	*-*
Normal weight	*n* = 128 (80.5%)	*n* = 95 (74.2%)	*n* = 50 (43.1%)
Overweight	*n* = 12 (7.5%)	*n* = 17 (13.3%)	*n* = 56 (48.3%)
Obese	*n* = 3 (1.9%)	*n* = 10 (7.8%)	*n* = 10 (8.6%)

**Table 3:** Frequencies of the International Physical Activity Questionnaire (IPAQ) categories.

IPAQ Children *n* = 123	Children	Mothers	Fathers
Low	-	2 (1.6%)	1 (.9%)
Moderate	98 (79.9%)	18 (14.5%)	12 (11.2%)
High	25 (20.4%)	104 (83.9%)	94 (87.9%)

**Table 4:** Dietary intake.

Difference from Recommended Amounts	Children *M* (*SD*)	Mothers *M* (*SD*)	Fathers *M* (*SD*)
Sweets (sweetened beverages, fast food, sweets, alcohol_parents_) Parents: 1750 kcal/week Children: 875 kcal/week	513.42 (1436.46)	−134.51 (1311.22)	979.87 (2209.31)
Fats and oil 14 portions/week	−4.79 (7.95)	−3.65 (8.86)	−2.66 (9.19)
Fish, meat, sausage and eggs 7 portions/week	0.27 (5.26)	0.64 (6.20)	3.61 (8.57)
Milk products 28 portions/week	−18.60 (6.90)	−19.38 (5.77)	−19.85 (7.70)
Wheat and potatoes 28 portions/week	−14.50 (7.24)	−13.96 (8.70)	−12.52 (10.09)
Fruits and vegetables 35 portions/week	−15.72 (16.27)	−17.57 (10.23)	−20.20 (9.72)
Water 10,500-mL/week	−3811.72 (3906.84)	−653.28 (4546.17)	−984.76 (5871.55)
